# More Medical Colleges

**Published:** 1879-11

**Authors:** 


MORE MEDICAL COLLEGES.—Last year the two medical colleges previously existing in Indianapolis, Indiana, were united into one; but in the October number of the *American Practitioner*, we are told that a second school has already been organized and is in operation in that city. It appears that a second medical school also exists in the ambitious city of Fort Wayne, which with the one at Evansville, makes five medical colleges in the State of Indiana; and yet only just enough students in the whole, to sustain one really good college.

We fully agree with the sentiment expressed in the following paragraph from the article alluded to in the *American Practitioner:* "A divorce between teaching medicine and the right to practice medicine, this journal advocated many years ago. It is one of the essential points in medical legislation. Such divorce obtained, and an honorable rivalry will exist among medical teachers to make thorough students instead of to get large classes—students who will pass with honor the rigid ordeal of a licensing board."

Such divorce we advocated many years before the *Practitioner* had an existence, and have continued to advocate it earnestly for more than thirty-five years; not only because it would make teachers and colleges rival each other in the extent and thoroughness of their instruction, but also because it would compel students to select and attend the colleges that gave the most thorough and complete instruction, instead of those whose fees and requirements were at the minimum end of the scale. D.

INTESTINAL OBSTRUCTIONS.—The clinical lecture that we copy from the *British Medical Journal*, in the present number of the JOURNAL AND EXAMINER, though headed "On Malignant Stricture," will be found to present a very full and interesting discussion of the diagnosis and treatment of intestinal obstructions, and will repay a careful perusal.

---

The statistics of suicides in France, just issued, show that nearly six thousand persons committed suicide last year.

